# Cardiovascular drug shortages in Lebanon: a broken heart

**DOI:** 10.1186/s13561-022-00369-9

**Published:** 2022-04-11

**Authors:** Georges Khattar, Jennifer Hallit, Carolla El Chamieh, Elie Bou Sanayeh

**Affiliations:** 1 Faculty of Medicine and Medical Sciences, Holy Spirit University of Kaslik (USEK), Jounieh, Lebanon; 2 Notre Dame des Secours University Hospital, Jbeil, Lebanon; 3 Department of Pediatrics, American University of Beirut Medical Center, Beirut, Lebanon; 4 Department of Internal Medicine, American University of Beirut Medical Center, Beirut, Lebanon

**Keywords:** Drug shortages, Pharmaceutical industry, Public health crisis

## Abstract

For nearly 3 years now, Lebanon has been assailed by compounded crises. With the economic instability, the coronavirus pandemic, and the explosion of the Beirut Port on August 4, 2020; the fragile Lebanese healthcare system has found itself at massive risk of a catastrophic public health crisis secondary to cardiovascular drug shortages. The time has come for public health authorities to find urgent solutions for this national trajedy that is projected to last for years.

## Background

Despite the enormous technological advances in the pharmaceutical industry and supply chains, drug shortages are a rising issue that peaked during the coronavirus-19 era, in the Middle East region [1] and specifically in Lebanon. Add to the effect of the pandemic, a drop in the local currency of more than 90% of its value with an enormous fall in the real Gross domestic product per capita of 37.1%; the fragile healthcare system in Lebanon has found itself at massive risk of a catastrophic public health crisis secondary to drug shortage [2].

### Cardiovascular drug shortages

As the leading cause of death worldwide and in Lebanon, cardiovascular diseases would be the pivot of the crisis. The shortages in essential outpatient cardiovascular drugs such as antihypertensives, antiplatelets, and antiarrhythmics have generated an increase in the incidence of decompensated heart failure, myocardial infarction secondary to stent thrombosis, and unstable arrhythmias requiring emergent interventions. Even in the inpatient setting, widely-available intravenous drugs have become extinct, such as loop diuretics, antiarrhythmics, anticoagulants, and even vasopressors, leaving physicians helpless in front of patients in life-threatening conditions. This situation has led to the loss of confidence in the Lebanese healthcare system despite its globally acclaimed hospitals, medical faculties, and internationally distinguished graduates [3].

### Root of the problem

Factors involved in drug shortages in Lebanon are complex. Following the Lebanese Civil War in 1975, the country’s healthcare system have been struggling to grow, mainly due to the multiple national and regional political conflicts and corruption. Therefore, this fragile system’s collapse was precipitated by the events that has been ongoing since 2019.

In a country that relies mainly on imported drugs, or imported raw materials for local production of some pharmaceutical products, the economic crisis with the shortage of foreign currency, the devaluation of the local one, and lifting subsidies on essential products have limited the importation and fabrication processes^4^. In fact, as per the World Bank, this economic and financial crisis ranks in the top 3 most severe crises globally since the nineteenth century, with an inflation rate of over 131.9% over the first 6 months of 2021 [2]. Moreover, the destruction of the country’s principal port in August 2020 has enormously aggravated the situation, leading to the failure of drugs’ production chains threatening the availability of any locally produced generic version. Subsequently, a massive strain was created on drug availability. Furthermore, as the government was planning to lift subsidies, fear of not finding chronic cardiovascular medications on the market has worsened the crisis as citizens rushed to hoarding them. Besides, smuggling subsidized cardiovascular drugs to foreign countries via black markets has created a considerable gap leaving the pharmacy shelves empty [4].

### Required interventions

Drug shortage in Lebanon is a national tragedy and a threat to national security that is projected to last for years, impacting the optimal medical management and the quality of care. Patients’ lives depend upon public health authorities to find urgent solutions for this critical healthcare issue. In addition to imposing strict control over the pharmaceutical business to disable black markets, potential solutions to guarantee continuous drugs availability would be to incentivize and sponsor local pharmaceutical companies to produce accessible alternatives, as importation options have become limited. Meanwhile, physicians must adapt to these strange circumstances and treat with what is available until a solution arises [5].

## Data Availability

Not applicable.
